# Regulation of the integrin αVβ3- actin filaments axis in early osteogenic differentiation of human mesenchymal stem cells under cyclic tensile stress

**DOI:** 10.1186/s12964-022-01027-7

**Published:** 2023-10-30

**Authors:** Yan Peng, Rongmei Qu, Yuchao Yang, Tingyu Fan, Bing Sun, Asmat Ullah Khan, Shutong Wu, Wenqing Liu, Jinhui Zhu, Junxin Chen, Xiaoqing Li, Jingxing Dai, Jun Ouyang

**Affiliations:** 1 Guangdong Provincial Key Laboratory of Medical Biomechanics and Guangdong Engineering Research Center for Translation of Medical 3D Printing Application and National Virtual and Reality Experimental Education Center for Medical Morphology and National Key Discipline of Human Anatomy, School of Basic Medical Sciences, Southern Medical University, Guangzhou, 510515 China; 2 Shenzhen Andy New Material Technology Co., LTD, Shenzhen, 518106 China

**Keywords:** Osteogenesis, Integrin αVβ3, Tensile stress, Mesenchymal stem cells, Yes-associated protein (YAP)

## Abstract

**Background:**

Integrins are closely related to mechanical conduction and play a crucial role in the osteogenesis of human mesenchymal stem cells. Here we wondered whether tensile stress could influence cell differentiation through integrin αVβ3.

**Methods:**

We inhibited the function of integrin αVβ3 of human mesenchymal stem cells by treating with c(RGDyk). Using cytochalasin D and verteporfin to inhibit polymerization of microfilament and function of nuclear Yes-associated protein (YAP), respectively. For each application, mesenchymal stem cells were loaded by cyclic tensile stress of 10% at 0.5 Hz for 2 h daily. Mesenchymal stem cells were harvested on day 7 post-treatment. Western blotting and quantitative RT-PCR were used to detect the expression of alkaline phosphatase (ALP), RUNX2, β-actin, integrin αVβ3, talin-1, vinculin, FAK, and nuclear YAP. Immunofluorescence staining detected vinculin, actin filaments, and YAP nuclear localization.

**Results:**

Cyclic tensile stress could increase the expression of ALP and RUNX2. Inhibition of integrin αVβ3 activation led to rearrangement of actin filaments and downregulated the expression of ALP, RUNX2 and promoted YAP nuclear localization. When microfilament polymerization was inhibited, ALP, RUNX2, and nuclear YAP nuclear localization decreased. Inhibition of YAP nuclear localization could reduce the expression of ALP and RUNX2.

**Conclusions:**

Cyclic tensile stress promotes early osteogenesis of human mesenchymal stem cells via the integrin αVβ3-actin filaments axis. YAP nuclear localization participates in this process of human mesenchymal stem cells.

Video Abstract

**Supplementary Information:**

The online version contains supplementary material available at 10.1186/s12964-022-01027-7.

## Introduction

Bone transport technology has been widely used in clinical practice since it was proposed by Ilizarov et al. [1]. It is one of the main methods for repairing limb bone defects. The theoretical basis of bone transfer is that under tensile-stress stimulation, such functions as cell proliferation, differentiation, biosynthesis, and tissue generation are more vigorous, thus promoting osteogenic differentiation by axial tensile stress and the intermittent mechanical stimuli of the osteotomy gap [2, 3].

An important direction of current research on how to repair large bone defects is how to promote the osteogenic differentiation of stem cells in vivo. Among others, mesenchymal stem cells (MSCs), including adipose-derived MSCs (ADSCs) and bone marrow MSCs (BMSCs), exist in the mesenchymal tissue of various organs in the body [4–6]. MSCs are pluripotent cells with osteogenic, chondrogenic, adipogenic, and myogenic differentiation functions. As such, MSCs have great potential in regenerative medicine [7, 8].

Integrins are transmembrane receptors on the cell surface and functional heterodimers composed of *α* subunits and *β* subunits through non-covalent bonding [9, 10]. Among others, 18 *α* subunits and eight *β* subunits can form 24 different heterodimers and bind to the extracellular matrix. Integrins form focal adhesion complexes by recruiting multiple intracellular proteins and connecting the intracellular microfilament cytoskeleton with the extracellular matrix (ECM), thus transmitting the mechanical signal from the outside to the inside of cells [11–14].

YAP is a transcriptional coactivator that binds to TEAD-DNA binding protein to promote cell proliferation and regulate stem cell differentiation [15–18]. However, the mechanism by which cyclic tensile stress (CTS) influences intracellular YAP expression and thereby regulates the osteogenesis of MSCs remains unclear.

In our previous studies, we explored the effect of cyclic tensile stress on human fibroblasts and found that cyclic tensile stress can promote the proliferation and osteogenesis of fibroblasts through integrin αVβ3 [19]. Here, our study showed the increased expression of integrin αVβ3 in cyclic tensile stress-induced osteogenesis of MSCs. Activation of integrin αVβ3, on the other hand, leads to the rearrangement of the actin filament structure and thereby regulates YAP nuclear localization. Ultimately, the mechanical signal influences the osteogenesis of MSCs through the αVβ3-microfilament axis. Our research indicates that αVβ3 may serve as a new target for bone repair at the level of stem cells.

## Materials and methods

### Chemicals and reagents

c(RGDyk) was purchased from Selleck (Shanghai, China) and dissolved in medium at concentration of 10 μM. RGD and RGE peptides were purchased from A-Peptide (Shanghai, China) and dissolved in medium at concentration of 10 μM. Cytochalasin D (Cyto D) and verteporfin (VP) were purchased from MedChemExpress (NJ, USA). Cytochalasin D dissolved in DMSO at concentration of 0.2 μg/mL and verteporfin dissolved in medium at concentration of 5 μM.

### Cell culture

BMSCs have been purchased from ScienCell research laboratories (San Diego, CA, USA, Cat #2320) and isolated from adult human bone marrow. ADSCs were isolated from adult human fat issues using the type I collagenase digestion method. We cultured MSCs using DMEM (Gibco, Grand Island, NY, USA) at 37 °C in a 5% CO_2_ atmosphere. 10% heat-inactivated fetal bovine serum (Gibco) and a 100 units/mL of penicillin (Gibco) and 100 μg/mL of streptomycin (Gibco) were added to the culture medium. The subsequent experiments used the fifth generation of cells.

After 48 h of cell passage, all inhibitors were applied for 24 h, followed by tensile stress. To maintain the working concentration during stretching, the medium including different kinds of inhibitors should be changed every 2 days.

Five main groups were established: undifferentiated MSCs grown in growth medium (GM group), MSCs applied by cyclic tensile stress for 7 days (CTS group), MSCs treated with RGDyk under cyclic tensile stress (RGDyk group), MSCs treated with cytochalasin D under cyclic tensile stress (CytoD group), and MSCs treated with verteporfin under cyclic tensile stress (VP group).

### Tensile stress loading

We planted MSCs on collagen I-coated silicone membrane plates (Flexcell International, NC, USA) at a density of 1 × 10^5^ cells per dish and cultured for 2 days before beginning experiments. using the Flexcell FX-5000 system (Flexcell International), cyclic tensile stress was applied to MSCs plated on six-well Bioflex Collagen I-coated plates. Flexcell FX-5000 system delivered a 10% tensile strain regimen at 0.5 Hz for 2 h each day. After applying cyclic tensile stress, MSCs used for ALP (alkaline phosphatase) staining, western blotting, qRT-PCR, and immunofluorescence were treated immediately. For comparative analysis, we used MSCs cultured under similar conditions but without cyclic stretch as unstretched controls.

### ALP staining

Wash the cells twice with phosphate-buffered saline (PBS) and fixed for 15 min with 4% paraformaldehyde on six-well Bioflex Collagen I-coated plates. After that, the cells were rinsed twice with PBS for 5 min and p-Nitrophenyl Phosphate as ALP reaction solution (Beyotime, Shanghai, China) was added. After incubation for 60 min at 37 °C, the cells were rinsed twice with PBS to remove the reaction solution. Images ALP staining were obtained directly by a camera (Honor-10 with Sony imx498, Huawei, China).

### RNA extraction and quantitative real-time PCR

Total cellular RNA of MSCs was extracted with Trizol lysis buffer. Qiagen (Shanghai, China) miRNA reverse transcription (miScript II RT Kit) and Thermo reverse transcription kits were used to transcribe RNA into cDNA. Using cDNA as the template, mRNA expression of integrin αV, integrin β3, ALP, RUNX2, Talin-1, FAK, vinculin, β-actin, and GAPDH were detected by quantitative real-time PCR (qRT-PCR). We used an ABI 7500 qRT-PCR system (Applied Biosystems) to perform qRT-PCR procedure. The specific primer sequences used are shown in Additional file 5: Table S1.

### Western blot analysis

After tensile stress loading, MSCs were harvested and washed twice by PBS. Cellular and nuclear protein was extracted by using radio-immunoprecipitation assay lysis buffer. Protein was isolated by 10% SDS polyacrylamide gel electrophoresis and transferred onto polyvinylidene difluoride (PVDF) membranes (Millipore, MMAS, USA). After electrophoresis, remove the PVDF membranes and seal it with 5% (w/v) skimmed milk powder for 1 h. All membranes were incubated with diluted primary antibody Additional file 6: (Table S2) at 4 °C overnight, using horseradish peroxidase-conjugated secondary antibody (1:5000, Beyotime) to wash and incubate at room temperature for 1 h. Enhanced chemiluminescence (ECL) chromogenic substrate enhanced immunoreactive protein bands and acquire images of bands.

### Immunofluorescence analysis

After tensile stress loading, MSCs were fixed in 4% paraformaldehyde solution for 20 min at room temperature immediately. Then, MSCs were immersed with 0.1% Triton X‐100 for 8 min at room temperature. After washing twice with PBS, MSCs were incubated with 5% bovine serum albumin/phosphate‐buffered saline (PBS) subsequently for 30 min. Samples were incubated overnight at 4℃ with primary antibody Additional file 7: (Table S3). After washing twice with PBS, cells were incubated with secondary antibody for 30 min in the dark. A confocal fluorescence microscope (LSM880, Zeiss, Germany) was used to view and acquire images. The LSM880 system used an oil immersion lens (63X, Numerical Aperture:1.4) and 568 nm laser wavelength to capture images.

### Statistical analysis

Statistical analysis was performed using SPSS 20.0 software (IBM, Inc. Armonk, NY, USA). All data are presented as mean ± SD of three independent experiments. Differences between the two groups were analyzed using the student’s *t*-test. A two-tailed value of *P* < 0.05 was considered statistically significant.

## Results

### Cyclic tensile stress promotes early-stage osteogenesis in MSCs

**Fig. 1:**
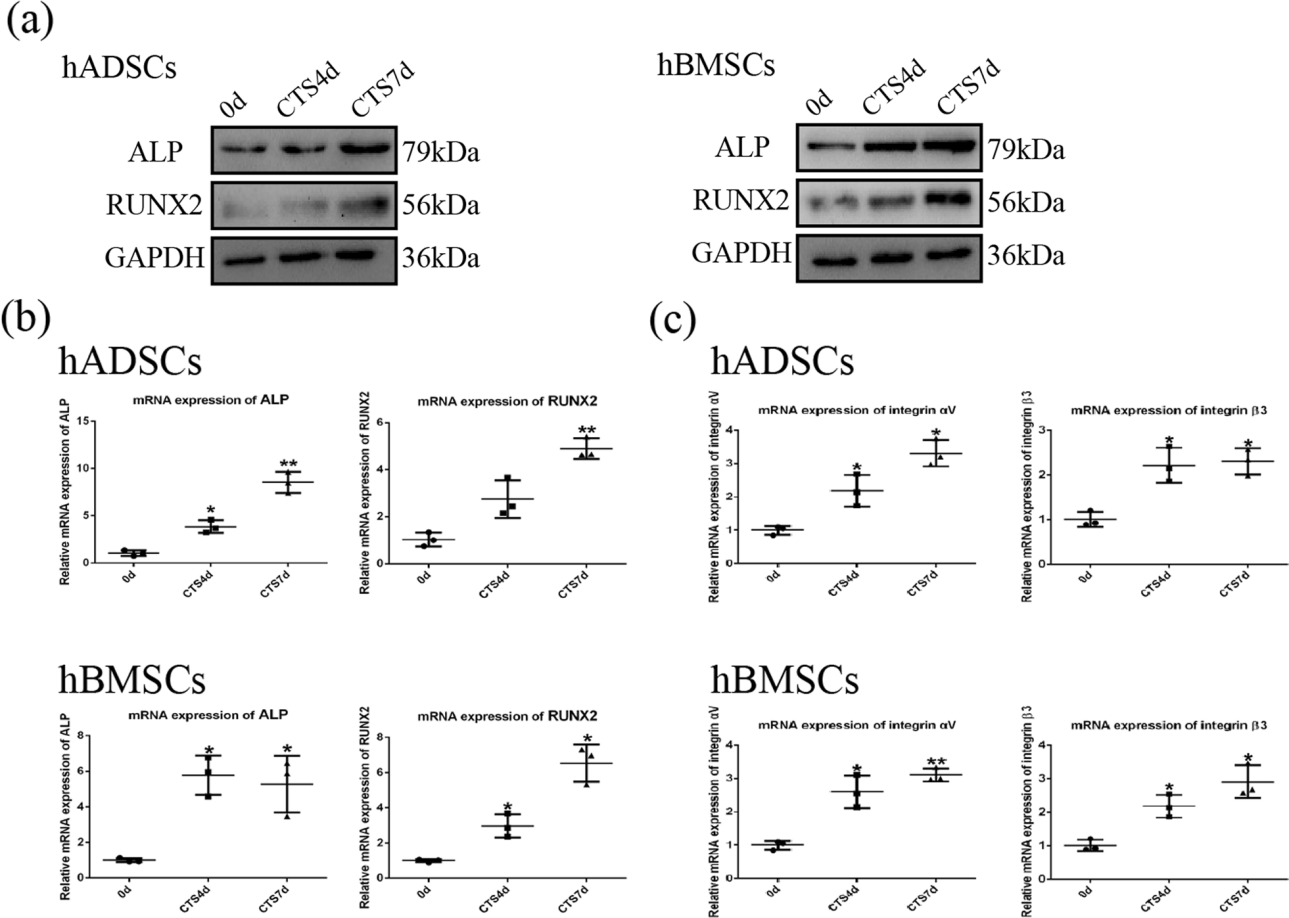
Early stage of osteogenic differentiation and expression of integrin αVβ3 in MSCs under CTS. **a** Protein expression of ALP and RUNX2 under CTS for 4 and 7 days. **b** mRNA expression of ALP and RUNX2 under CTS for 4 and 7 days. **c** mRNA expression of integrin αVβ3 under CTS for 4 and 7 days. (Symbol meaning in each statistical graph: Vertical Lines represents the average of three groups of data; Error Bars represents standard deviation; * represents *P* < 0.05, ** represents *P* < 0.01. All statistical analyses were performed between the experimental group and control group.)

**Fig. 2:**
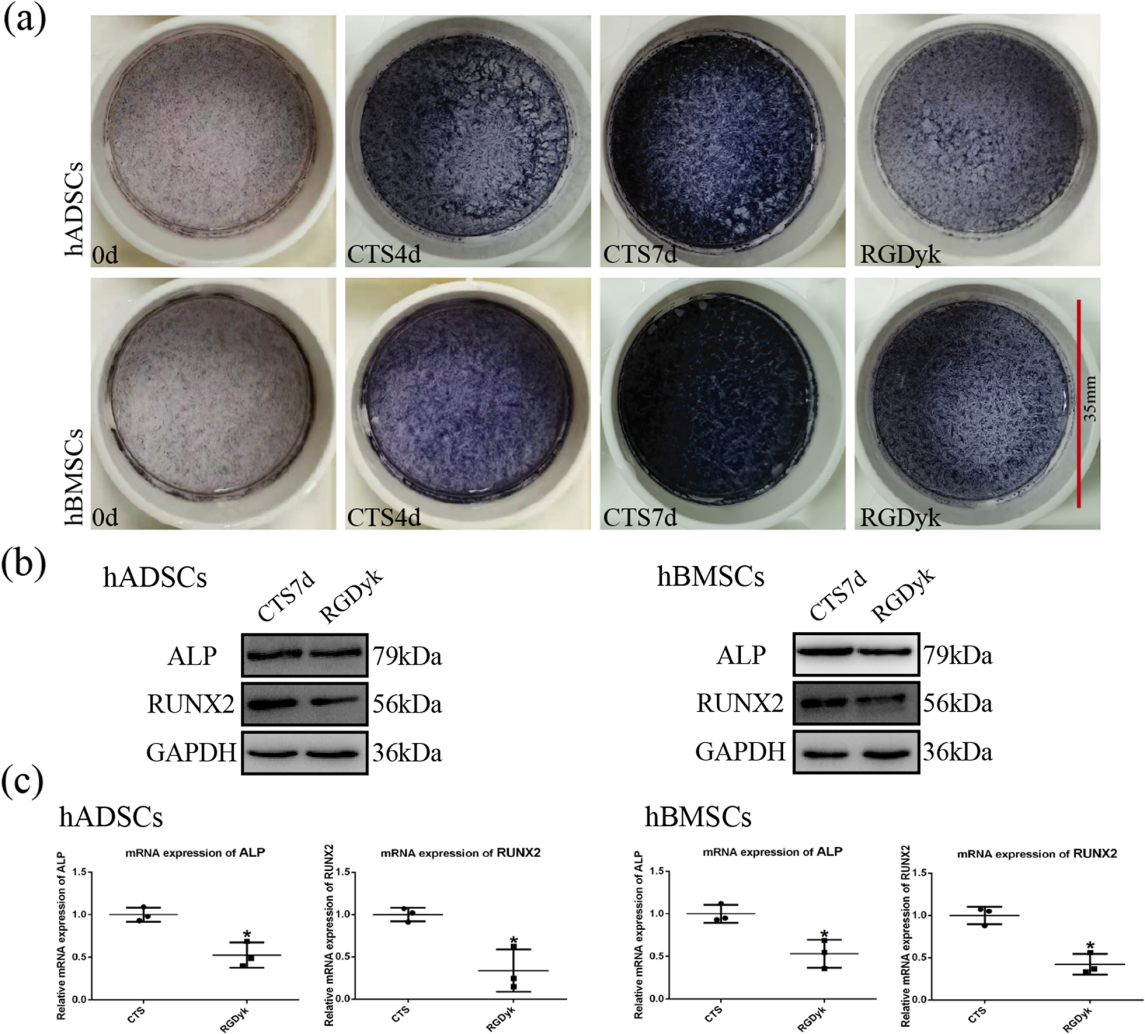
CTS regulated early stage of osteogenic differentiation of MSCs via integrin αVβ3. **a** ALP staining under CTS in the GM, CTS, and RGDyk groups. **b** Protein expression of ALP and RUNX2 under CTS in the GM and RGDyk groups. **c** mRNA expression of ALP and RUNX2 under CTS in the GM and RGDyk groups. (Symbol meaning in each statistical graph: Vertical Lines represents the average of three groups of data; Error Bars represents standard deviation; * represents *P* < 0.05, ** represents *P* < 0.01. All statistical analyses were performed between the experimental group and control group.)

Our study investigated the relationship between cyclic tensile stress and early-stage osteogenic differentiation in MSCs. Compared with the control group, we observed an increase in protein and mRNA expression of the osteogenic markers ALP and RUNX2 in MSCs after 7 days of cyclic tensile stress loading (Fig. 1a, b). The corresponding results were observed for ALP staining of MSCs. The ALP staining of MSCs was significantly deepened under cyclic tensile stress (Fig. 2a). These results suggest that we can promote the early osteogenic differentiation of MSCs by cyclic tensile stress.

### Early-stage osteogenic differentiation induced by cyclic tensile stress depends on the expression and activation of integrin αVβ3

To investigate whether integrins are involved in the osteogenic differentiation of MSCs induced by cyclic tensile stress, we analyzed the expression level of integrin αVβ3 by qRT-PCR. We found that the expression of integrin αVβ3 under cyclic tensile stress was higher than that in control group, suggesting that cyclic tensile stress promoted integrin αVβ3 expression (Fig. 1c). Therefore, we studied the relationship between integrin αVβ3 and early-stage osteogenic differentiation of MSCs induced by cyclic tensile stress. By RGDyk, an antagonist of integrin αVβ3, we inhibited the function of integrin αVβ3 in MSCs (Additional file 1: Figure S1).

The ALP staining in the RGDyk group was shallow compared to the CTS group (Fig. 2a). And western blotting showed that the expression of osteogenic markers ALP and RUNX2 was reduced in the RGDyk group relative to the CTS group when MSC integrin αVβ3 was inhibited (Fig. 2b). The qRT-PCR assay also showed similar results. When the integrin αVβ3 function was inhibited, the mRNA expression of both ALP and RUNX2 was decreased (Fig. 2c). The above results indicate that cyclic tensile stress can affect the early osteogenic differentiation of MSCs through integrin αVβ3.

### Cyclic tensile stress promoted the formation of focal adhesions during osteogenesis

**Fig. 3:**
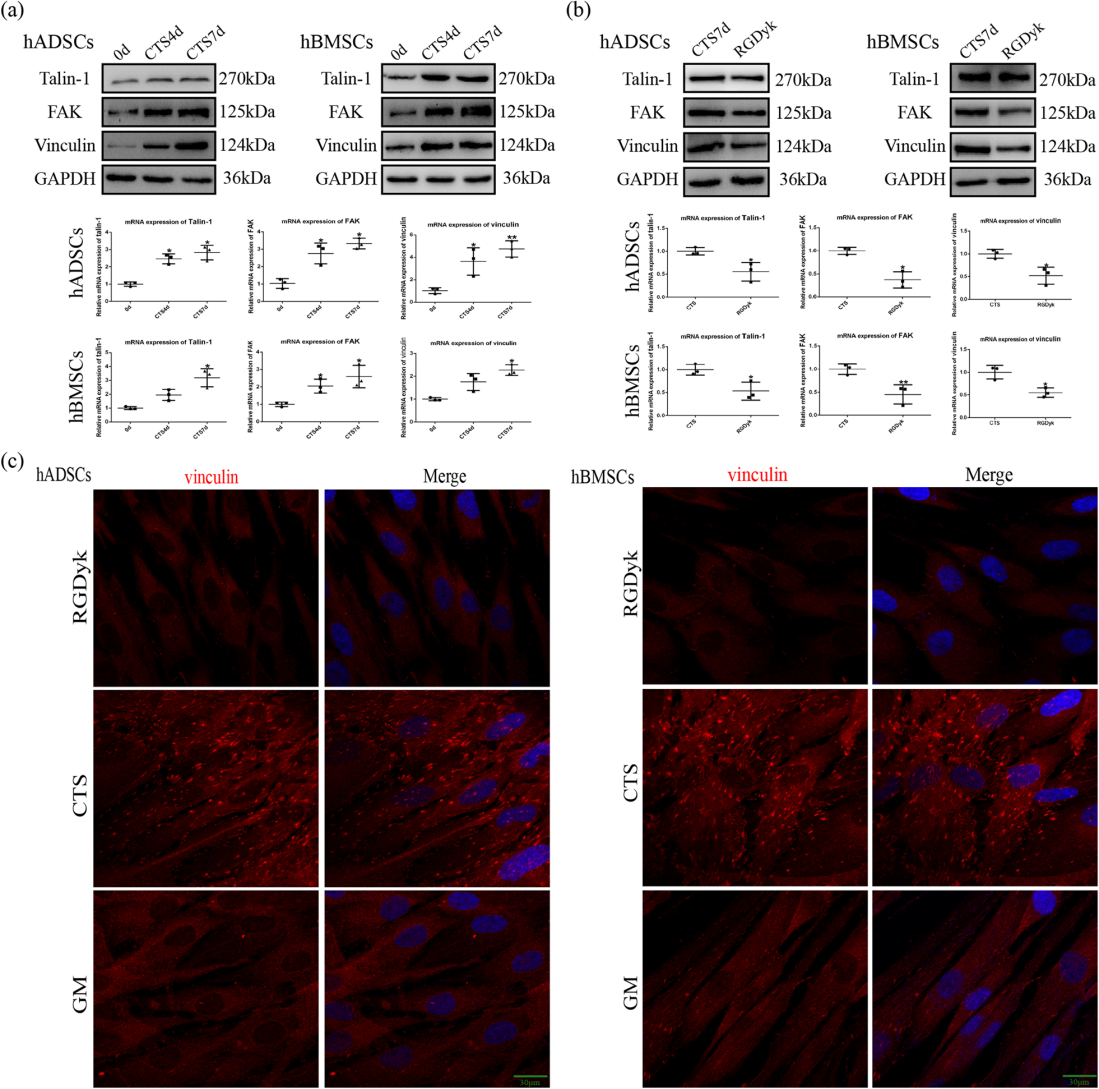
Integrin αVβ3 activation induced by CTS regulates focal adhesion-related proteins to promote early stage of osteogenic differentiation. **a** Protein and mRNA expression of talin-1, FAK, and vinculin under CTS for 4 and 7 days. **b** Protein and mRNA expression of talin-1, FAK, and vinculin under CTS in the CTS and RGDyk groups. **c** Immunofluorescence of vinculin under CTS in the GM, CTS, and RGDyk groups for 7 days. Scale bar, 20 μm. (Symbol meaning in each statistical graph: Vertical Lines represents the average of three groups of data; Error Bars represents standard deviation; * represents *P* < 0.05, ** represents *P* < 0.01. All statistical analyses were performed between the experimental group and control group.)

The focal adhesion complex connects integrin αVβ3 and actin filaments and uses this to conduct mechanical signals. We explored the expression of talin-1, FAK, and vinculin under cyclic tensile stress and examined the expression of talin-1, FAK, and vinculin under integrin αVβ3 activation. Western blotting and qRT-PCR results showed that cyclic tensile stress effectively increased talin-1, FAK, and vinculin expression levels in MSCs which compared with the control group (Fig. 3a). After we inhibited integrin αVβ3 function using RGDyk, western blotting showed that the expression of talin-1, FAK, and vinculin proteins in the RGDyk group decreased relative to the CTS group. The qRT-PCR experiments yielded similar results (Fig. 3b).

Immunofluorescence showed that vinculin staining in the GM group clearly followed the cell outline, and a small number of highlighted spots representing the focal adhesion complex were visible.

In contrast, the highlight spots in their vinculin staining significantly increased under cyclic tensile stress. When integrin αVβ3 function was inhibited, the number of high-bright spots was significantly reduced (Fig. 3c). The above results suggest that cyclic tensile stress increases focal adhesion formation in MSCs by activating integrin αVβ3.

### Cyclic tensile stress-regulated actin filaments via integrin αVβ3 during osteogenesis

**Fig. 4:**
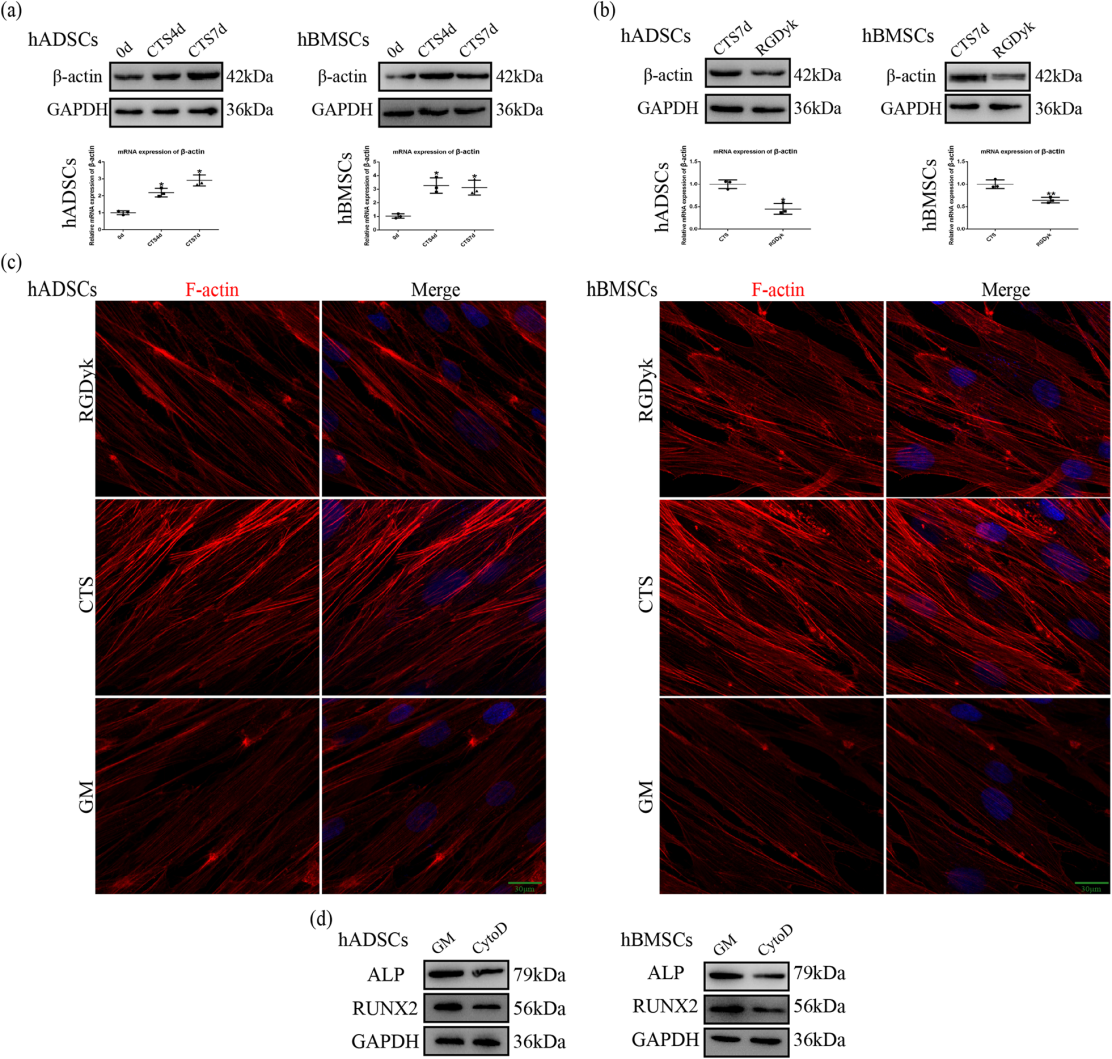
Actin filaments regulated early stage of osteogenic differentiation induced by CTS via integrin αVβ3 activation in MSCs. **a** Protein and mRNA expression of β-actin under CTS for 4 and 7 days. **b** Protein and mRNA expression of β-actin under CTS in the CTS and RGDyk groups. **c** Immunofluorescence of F-actin under CTS in the GM, CTS, and RGDyk groups for 7 days. Scale bar, 20 μm. **d** Protein expression of ALP and RUNX2 under CTS in the GM and CytoD groups. (Symbol meaning in each statistical graph: Vertical Lines represents the average of three groups of data; Error Bars represents standard deviation; * represents *P* < 0.05, ** represents *P* < 0.01. All statistical analyses were performed between the experimental group and control group.)

Through western blotting and qRT-PCR experiments, we found that the expression of β-actin was elevated under the effect of cyclic tensile stress (Fig. 4a). To understand the relationship between integrin αVβ3 and actin filaments during mechanotransduction, we examined the changes in β-actin expression after functional inhibition of integrin αVβ3. The results showed that the level of β-actin expression in the RGDyk group in MSCs was reduced compared with that in the CTS group (Fig. 4b). Immunofluorescence experiments showed that actin filaments in the GM group were elongated, evenly aligned, and parallel. Under cyclic tensile stress, actin stress filaments became thicker, shorter, and clustered toward the cell membrane. When integrin αVβ3 function was inhibited, the actin stress filaments were slightly thickened and arranged in a uniform and orderly manner similar to the GM group (Fig. 4c). The above results suggest that cyclic tensile stress regulates β-actin expression through integrin αVβ3 and affects the structure of actin filaments.

Next, to study the relationship between actin filaments and osteogenic differentiation of MSCs induced by cyclic tensile stress, we used cytochalasin D to inhibit the aggregation of actin filaments (Additional file 2: Figure S2a). Western blotting results showed that cytochalasin D reduced the expression of ALP and RUNX2 under tensile stress (Fig. 4d). The results suggest that cyclic tensile stress can promote early-stage osteogenic differentiation of MSCs via the integrin αVβ3-actin filaments axis.

### Cyclic tensile stress-regulated YAP nuclear localization via the integrin αVβ3-actin filaments axis during osteogenesis

**Fig. 5:**
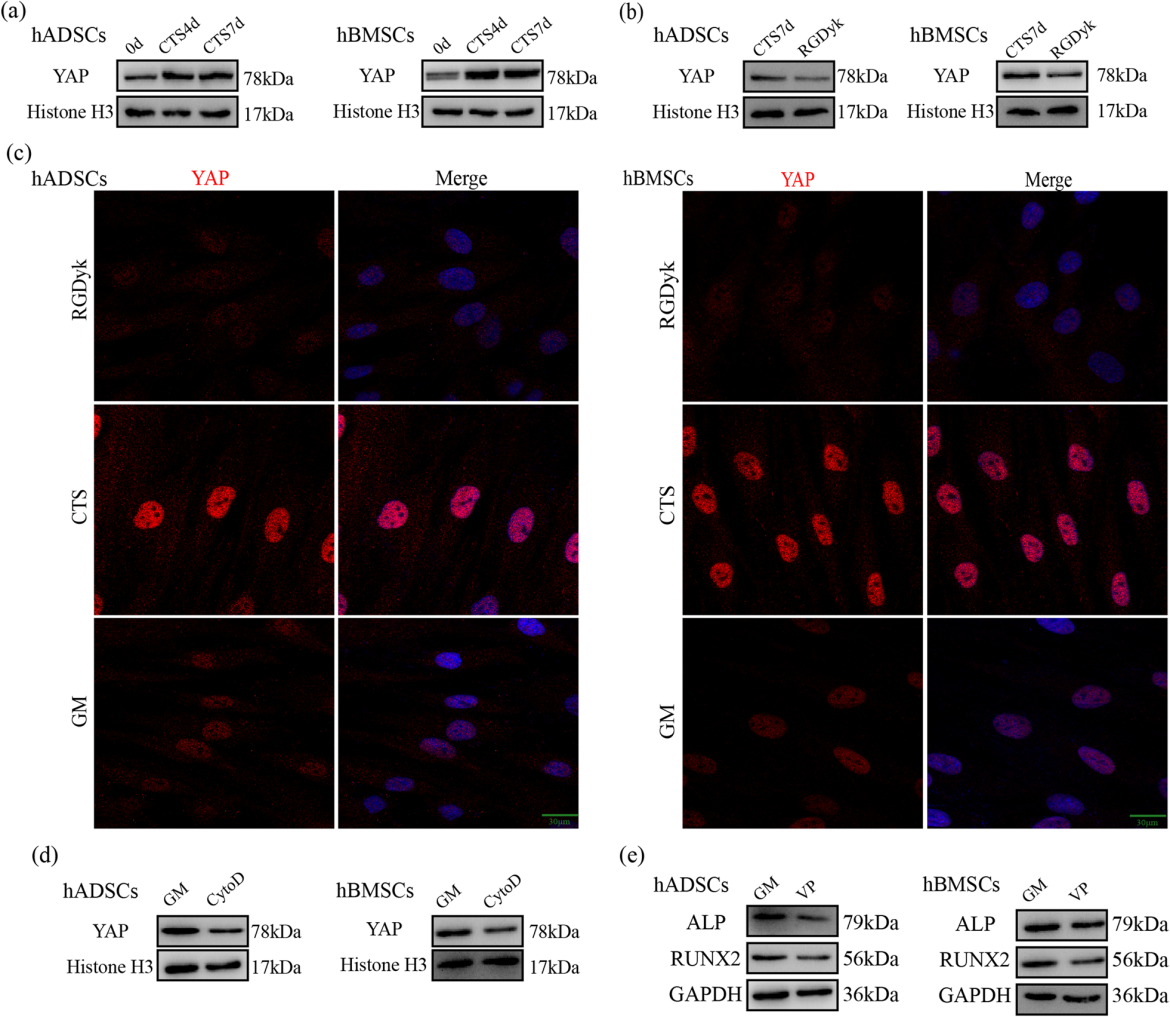
Integrin αVβ3 and actin filaments regulate expression of nuclear YAP during early stage of osteogenic differentiation induced by CTS. **a** Protein expression of nuclear YAP under CTS for 4 and 7 days. **b** Protein expression of nuclear YAP under CTS in the CTS and RGDyk groups. **c** Immunofluorescence of YAP under CTS in the GM, CTS, and RGDyk groups for 7 days. Scale bar, 20 μm. **d** Protein expression of nuclear YAP under CTS in the GM and CytoD groups. **e** Protein expression of ALP and RUNX2 under CTS in the GM and VP groups

In addition, our study explored the mechanism of cyclic tensile stress on nuclear YAP. Western blotting showed that cyclic tensile stress increased the expression of nuclear YAP (Fig. 5a). After the function of integrin αVβ3 was inhibited, western blotting presented that the expression of nuclear YAP in the RGDyk group was lower than that in the CTS group (Fig. 5b). According to the immunofluorescence results, the stained nuclear YAP in the GM group presented a regular nuclear outline. Under cyclic tensile stress, the staining of nuclear YAP was significantly deepened. After functional inhibition of integrin αVβ3, nuclear YAP staining in the RGDyk group was lower than in the CTS group (Fig. 5c). The above results suggest that cyclic tensile stress affects YAP nuclear localization through integrin αVβ3.

When β-actin polymerization was inhibited by cytochalasin D, western blotting showed that the expression of nuclear YAP decreased under cyclic tensile stress (Fig. 5d). Further, the function of nuclear YAP was inhibited using verteporfin (Additional file 2: Figure S2b), and western blotting showed that YAP nuclear localization was reduced under tensile stress (Fig. 5e). These findings suggest that promoting cyclic tensile stress on actin filament structure can increase YAP nuclear localization, thus affecting the early osteogenic differentiation of MSCs.

## Discussion

During bone transport, the bones and tissues in the body are subjected to mechanical force. These mechanical signals affect the structure of bones and the differentiation direction of cells in the body through the signal transduction pathway [20–22]. Integrins are an important mechanical receptor in cell response to external mechanical loading [23–26]. In the present study, we found that integrin αVβ3 played a role in the early osteogenic differentiation of MSCs induced by cyclic tensile stress.

Although different researchers have adopted different mechanical parameters, previous studies based on the FLEXCELL-5000 tension system have proposed that tensile stress can induce the osteogenic differentiation of MSCs [19, 27–29]. Our experiments also verified that cyclic tensile stress could promote the early osteogenic differentiation of MSCs without chemical osteogenic inducers. However, the correlation between cyclic tensile stress and the direction of stem cell differentiation needs to be further explored. Integrins can connect cells with the extracellular matrix, the cell membrane, cytoskeleton and activate multiple intracellular signal transduction pathways, which provides a mechanical basis for mechanical conduction. It has been shown that integrin αVβ3 mediates the expression of sclerostin through periostin, thereby promoting osteogenesis and survival under mechanical stimulation [30]. In addition, integrin αVβ3 activation by the mechanical load can up-regulate the expressions of c-fos, IGF-1, and COX-2, thus affecting the calcium influx of osteoblasts [31]. Our results also show that cyclic tensile loading increased the expression of integrin αVβ3. In as next step we focused of the role of integrin αVβ3 in the early osteogenic induction of MSCs under tensile stress. (Additional file 3).

Integrins-based focal adhesion complexes are formed during mechanical loading between cells and the extracellular matrix. The activation of integrins recruited proteins such as vinculin, FAK, and talin to form focal adhesion complexes [32–35]. Our study found that cyclic tensile stress recruited intracellular proteins to form focal adhesion complexes through integrin αVβ3 and conduct mechanical signals through focal adhesion complexes. Therefore, we believe that during cyclic tensile stress loading, focal adhesion complexes formation is concentrated in the adhesion sites between cells and the extracellular matrix so that cells can better adapt to the mechanical microenvironment and produce directional differentiation. (Additional file 4).

The microfilament cytoskeleton formed by actin polymerization plays a unique role in the early-stage osteogenic differentiation of MSCs. The arrangement of the microfilament cytoskeleton of MSCs changes during osteogenic differentiation. When stem cells differentiate into osteoblasts, the microfilament cytoskeleton becomes more dispersed and larger with the differentiation of MSCs [36–39]. Our study showed that the formation of actin filaments increased under cyclic tensile stress, which regulated the osteogenic differentiation of MSCs. Through the immunofluorescence results, we speculate that the actin stress filaments polymerized by actin converge to the cell membrane to connect and form increased focal adhesion complexes and the thickening of actin stress filaments makes MSCs better adapt to mechanical loading and conduct mechanical signals. Additionally, the response of nuclear YAP by mechanical load also requires participation of microfilament cytoskeleton. It has been demonstrated that actin polymerization can stimulate YAP nuclear localization. Knockout of actin capping protein can increase actin polymerization to form more microfilaments, increasing YAP/TAZ nuclear localization [40–42]. A recent study has shown that β-catenin signaling pathway regulates the expression of YAP during osteogenic differentiation [43]. Our study demonstrated that under cyclic tensile stress, the activation of the integrin αVβ3-actin filament axis could regulate YAP nuclear localization. Therefore, we believe that YAP protein plays different roles in multiple signaling pathways related to osteogenic differentiation.

For a long time, researchers have been committed to explore the specific mechanism of physical factor inducing osteogenic differentiation of MSCs. Lin er al. reported that anisotropic magneto-mechanical stimulation activates integrin α5β1 when it causes matrix stretching, and integrin αVβ3 when it causes matrix bending. The different type of anisotropic Magneto-mechanical stimulation causes different deformation of the cytoskeleton and this results in nuclear localization of YAP/TAZ and promotes osteogenic differentiation [44]. Lee er al. reported that geometry of matrix guides the spatial positioning of focal adhesions and reveal how mechanical forces influence osteogenic differentiation of MSCs through integrin αVβ3 and α5β1 [45]. Previous studies have demonstrated the important role of integrin αVβ3 in mechanical conduction and suggested that integrin αVβ3 activation can affect intracellular cytoskeletal architecture. In our previous study, cyclic tensile stress transmitted mechanical signals through integrin αVβ3 and actin filaments to promote osteogenesis of fibroblasts. Currently, similar results in MSCs lead us to speculate that mechanical forces activate intracellular signaling pathways through similar sites in different cells, which have the potential to differentiate. Whether different types of mechanical forces promote specific stem cells differentiation at different sites requires more detailed research in the future. Even under the same mechanical force, the response of different cells to the force is not consistent. Compared with the actin filaments structure of MSCs, the actin filaments structure of fibroblasts changed more dramatically by tensile stress. Considering the difference in osteogenic differentiation potential between the fibroblasts and MSCs, we believe that MSCs with better osteogenic potential may be more easily activated through osteogenic signaling pathways by tensile stress, while fibroblasts need more drastic changes to adapt to the new mechanical microenvironment.

The experimental results of this study contribute to a new understanding of the early osteogenic differentiation of human MSCs induced by cyclic tensile stress. Cyclic tensile stress positively affects the early osteogenic differentiation of MSCs through the activation of integrin αVβ3, resulting in the structural changes of intracellular actin filaments. By activating integrin αVβ3, the structural changes of actin filaments under the stimulation of cyclic tensile stress led to increased YAP nuclear localization. Therefore, integrin αVβ3 and YAP nuclear localization can participate in the early osteogenic differentiation of MSCs. In future research on bone repair, integrin αVβ3 should be considered a potential experimental target in vivo.

## Conclusion

Cyclic tensile stress positively affects the early stage of osteogenic differentiation of MSCs via integrin αVβ3 activation, leading to rearrangement of the actin filaments and increased formation of focal adhesion complexes. Furthermore, rearrangement of actin filaments stimulated by cyclic tensile stress leads to increased YAP nuclear localization via integrin αVβ3 in human MSCs. YAP nuclear localization also assisted in cyclic tensile stress promoting osteogenic differentiation of MSCs.

### Supplementary Information

**Additional file 1: Fig. S1. **The function of integrin αVβ3 was inhibited by c(RGDyk) (10 μM). Scale bar, 20 μm.

**Additional file 2: Fig. S2. a** Polymerization of β-actin was inhibited by cytochalasin D (0.2 μg/mL). **b** The function of nuclear YAP was inhibited by verteporfin (5 μM).

**Additional file 3: Fig. S3. **Images of western blots.

**Additional file 4: Fig. S3. **ALP staining of hADSCs and hBMSCs in growth medium for 4 and 7 days. ALP staining was obtained directly by a camera (Reno 8 with Sony imx766, OPPO, China).

**Additional file 5**

**Additional file 6**

**Additional file 7**

## Data Availability

All the supporting data can be downloaded.

## Abbreviations

ALP: Alkaline phosphatase
Cyto D: Cytochalasin D
CST: Cyclic tensile stress
ECM: Extracellular matrix
ECL: Enhanced chemiluminescence
FAK: Focal adhesion kinase
GAPDH: Glyceraldehyde-3-phosphate dehydrogenase
GM: Growth medium
PBS: Phosphate-buffered saline
PVDF: Polyvinylidene difluoride
RUNX2: Runt-related transcription factor 2
TEAD: TEA domain DNA-binding family of transcription factors
VP: Verteporfin
YAP: Yes-associated protein
