# Supplementary figures and images for: Regulation of the integrin αVβ3- actin filaments axis in early osteogenic differentiation of human mesenchymal stem cells under cyclic tensile stress
Source: Cell Commun Signal. 2023 Oct 30;21:308. doi: 10.1186/s12964-022-01027-7 (PMC10614380; doi:10.1186/s12964-022-01027-7)

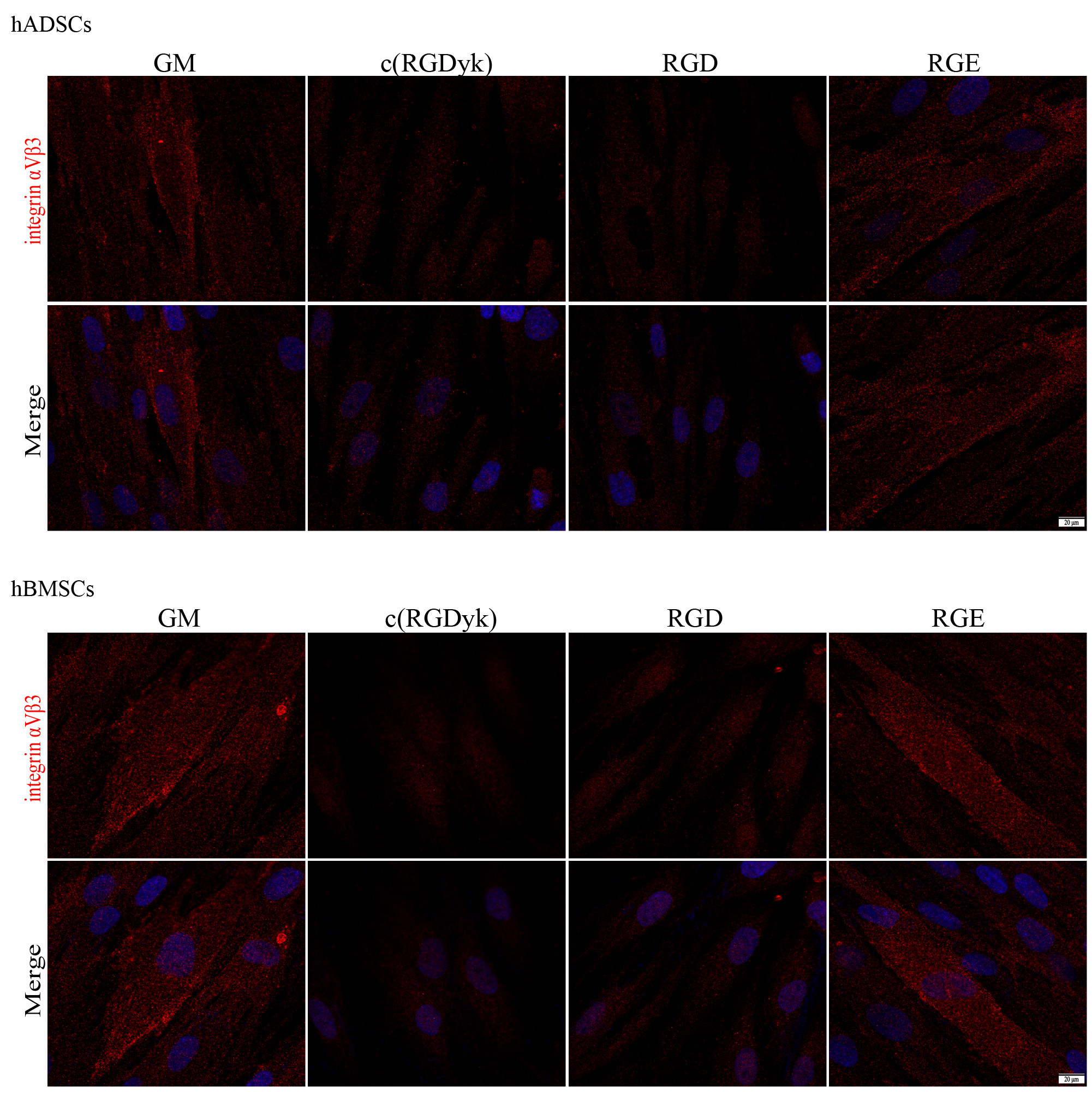

Supplement: Supplementary file 2 — Additional file 1: Fig. S1. The function of integrin αVβ3 was inhibited by c(RGDyk) (10 μM). Scale bar, 20 μm. [file 12964_2022_1027_MOESM1_ESM.tif]

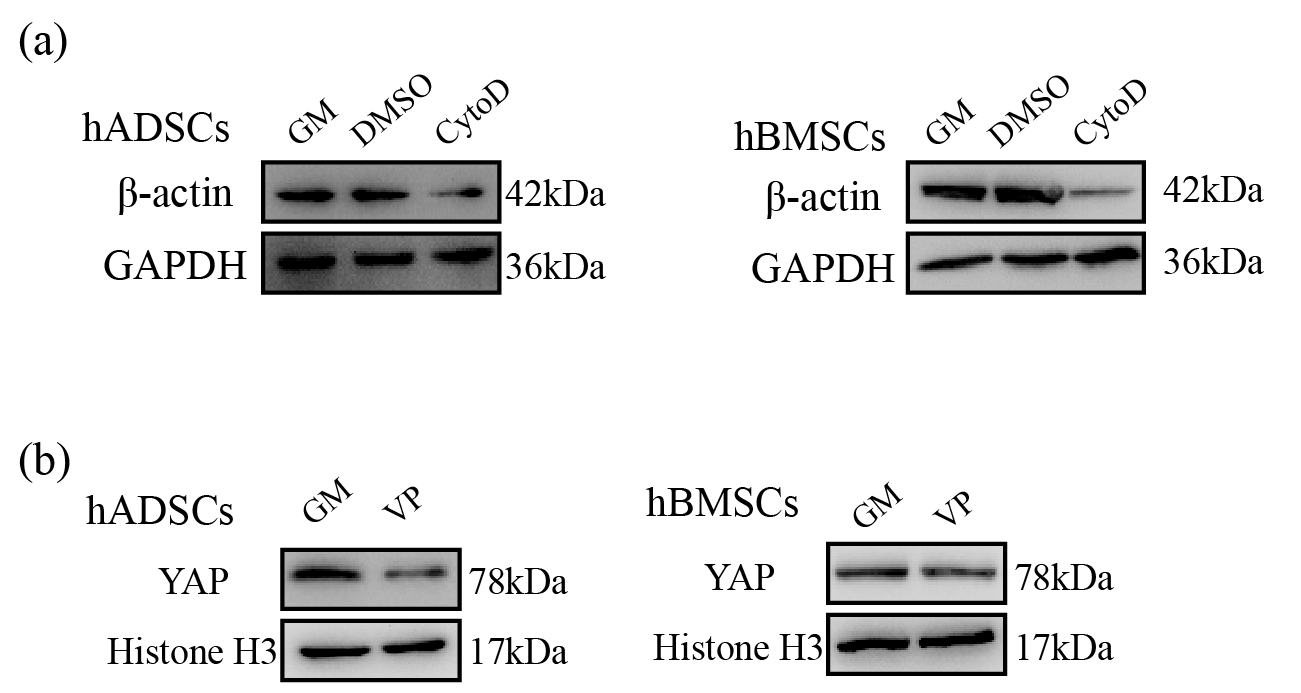

Supplement: Supplementary file 3 — Additional file 2: Fig. S2. a Polymerization of β-actin was inhibited by cytochalasin D (0.2 μg/mL). b The function of nuclear YAP was inhibited by verteporfin (5 μM). [file 12964_2022_1027_MOESM2_ESM.tif]

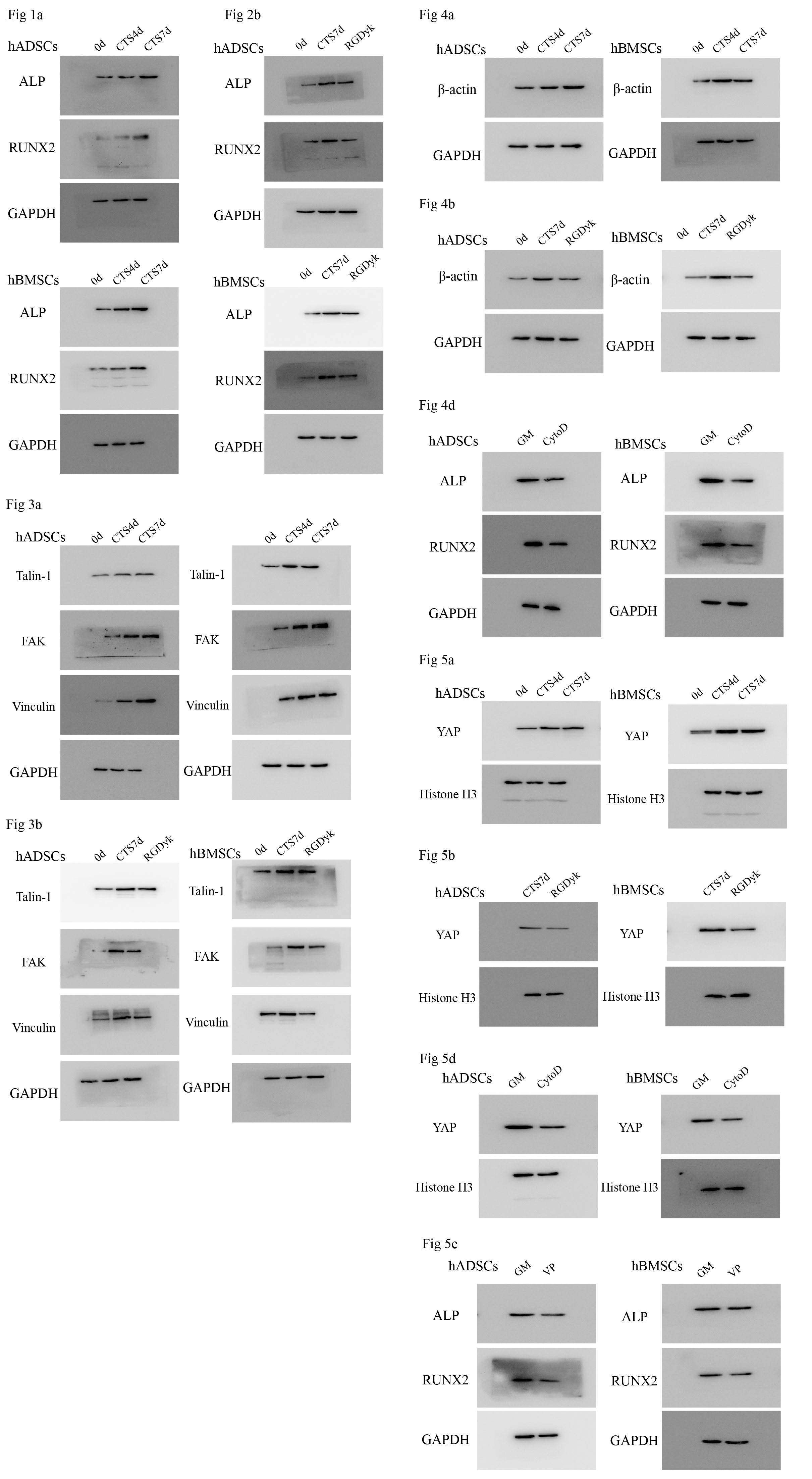

Supplement: Supplementary file 4 — Additional file 3: Fig. S3. Images of western blots. [file 12964_2022_1027_MOESM3_ESM.tif]

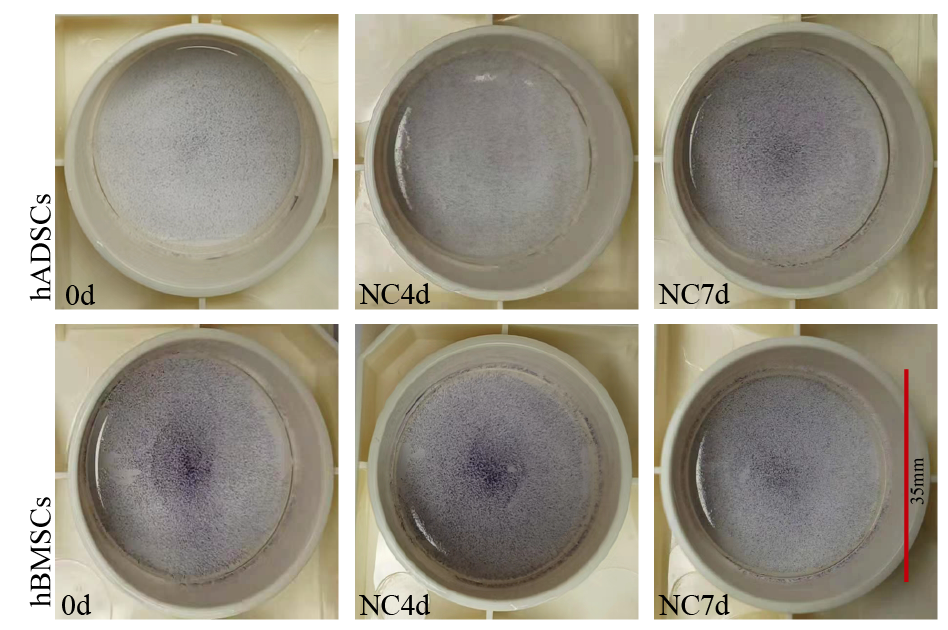

Supplement: Supplementary file 5 — Additional file 4: Fig. S3. ALP staining of hADSCs and hBMSCs in growth medium for 4 and 7 days. ALP staining was obtained directly by a camera (Reno 8 with Sony imx766, OPPO, China). [file 12964_2022_1027_MOESM4_ESM.tif]
