# Supplementary material for: Regulation of the integrin αVβ3- actin filaments axis in early osteogenic differentiation of human mesenchymal stem cells under cyclic tensile stress
Source: Cell Commun Signal. 2023 Oct 30;21:308. doi: 10.1186/s12964-022-01027-7 (PMC10614380; doi:10.1186/s12964-022-01027-7)
Supplement: Supplementary file 6 — Additional file 5 [file 12964_2022_1027_MOESM5_ESM.docx]

Table S1. Primers used in the qRT-PCR.

| Gene | Forward primer | Reverse primer |
| --- | --- | --- |
| GAPDH | TCGGAGTCAACGGATTTGGT | TTCCCGTTCTCAGCCTTGAC |
| RUNX2 | GAGATCATCGCCGACCAC | TACCTCTCCGAGGGCTACC |
| ALP | ACCATTCCCACGTCTTCACATTTG | AGACATTCTCTCGTTCACCGCC |
| ITG AV | CCGAAGCTCAGCCCTCTTG | GAAAAGCCATCGCCGAAGTG |
| ITG B3 | ACCAGTAACCTGCGGATTGG | CTCATTGAAGCGGGTCACCT |
| Talin-1 | GGAAAAGTTGCGGGGCATAG | CAAGAACACAGGCCGTTTGG |
| FAK | CAGGGTCCGATTGGAAACCA | CTGAAGCTTGACACCCTCGT |
| vinculin | CGCTGAGGTGGGTATAGGTG | GTAGCTTCCCGATGCAAGGA |
