# Supplementary material for: Regulation of the integrin αVβ3- actin filaments axis in early osteogenic differentiation of human mesenchymal stem cells under cyclic tensile stress
Source: Cell Commun Signal. 2023 Oct 30;21:308. doi: 10.1186/s12964-022-01027-7 (PMC10614380; doi:10.1186/s12964-022-01027-7)
Supplement: Supplementary file 7 — Additional file 6 [file 12964_2022_1027_MOESM6_ESM.docx]

Table S3 Primary antibodies/dye used in immunofluorescence.

| Antibody/dye | Company |
| --- | --- |
| mouse anti-integrin αVβ3 | 1:200, Millipore, Cat # MAB1976 |
| rabbit anti-YAP | 1:200, Cell Signaling Technology |
| Phalloidin Red-594 | 1:500, Beyotime, Cat # C2203S |
| mouse anti-vinculin | 1:200, Sigma |
