# Supplementary material for: Regulation of the integrin αVβ3- actin filaments axis in early osteogenic differentiation of human mesenchymal stem cells under cyclic tensile stress
Source: Cell Commun Signal. 2023 Oct 30;21:308. doi: 10.1186/s12964-022-01027-7 (PMC10614380; doi:10.1186/s12964-022-01027-7)
Supplement: Supplementary file 8 — Additional file 7 [file 12964_2022_1027_MOESM7_ESM.docx]

Table S2 Primary antibodies used in western blotting

| Antibody | Company |
| --- | --- |
| rabbit anti-RUNX2 (runt-related transcription factor 2) | 1:1000 Cell Signaling Technology, Cat # 12556S |
| mouse anti-ALP | 1:1000, Abcam, Cat # AB126820 |
| rabbit anti-β-actin | 1:1000, Cell Signaling Technology, Cat # 4970S |
| mouse anti-vinculin | 1:1000, Sigma, Cat # V9131 |
| rabbit anti-talin1 | 1:1000, Cell Signaling Technology, Cat #4021S |
| mouse anti-FAK (focal adhesion kinase) | 1:500, Santa Cruz, Cat # SC1688 |
| rabbit anti-YAP | 1:1000, Cell Signaling Technology, Cat # 14074S |
| rabbit anti-GAPDH | 1:5000, Bioworld, Cat # AP0063 |
